# *KRAS*, a New Target for Precision Medicine in Colorectal Cancer?

**DOI:** 10.3390/cancers16203455

**Published:** 2024-10-12

**Authors:** Alice Boilève, Cristina Smolenschi, Aurélien Lambert, Valérie Boige, Matthieu Delaye, Géraldine M. Camilleri, Anthony Tarabay, Marine Valéry, Alina Fuerea, Thomas Pudlarz, Jacques R. R. Mathieu, Fanny Jaulin, Antoine Hollebecque, Michel Ducreux

**Affiliations:** 1 Gustave Roussy, Département de Médecine Oncologique, 94805 Villejuif, France; cristina.smolenschi@gustaveroussy.fr (C.S.); valerie.boige@gustaveroussy.fr (V.B.); matthieu.delaye@gustaveroussy.fr (M.D.); geraldine.camilleri@gustaveroussy.fr (G.M.C.); anthony.tarabay@gustaveroussy.fr (A.T.); marine.valery@gustaveroussy.fr (M.V.); alinacristina.fuerea@gustaveroussy.fr (A.F.); thomas.pudlarz@gustaveroussy.fr (T.P.); antoine.hollebecque@gustaveroussy.fr (A.H.); michel.ducreux@gustaveroussy.fr (M.D.); 2 Université Paris-Saclay, 91400 Orsay, France; jacques.mathieu@gustaveroussy.fr (J.R.R.M.); fanny.jaulin@gustaveroussy.fr (F.J.); 3 Gustave Roussy, Unité INSERM U1279, 94805 Villejuif, France; 4 Gustave Roussy, Département d’Innovation Thérapeutiques et d’Essais Précoces, 94805 Villejuif, France; 5 Institut de Cancérologie de Lorraine, INSERM, INSPIIRE, Université de Lorraine, 54052 Nancy, France; a.lambert@nancy.unicancer.fr

**Keywords:** colorectal cancer, precision medicine, *KRAS*, molecular profile, targeted therapies

## Abstract

**Simple Summary:**

Colorectal cancer (CRC) is a deadly disease in which *KRAS* mutations are prevalent and are associated with poor prognosis. The emergence of KRAS inhibitors is a promising treatment option. This review discusses various classes of KRAS inhibitors, that can be used alone or combined to overcome resistance mechanisms. It highlights recent clinical trials evaluating the efficacy of various strategies to target KRAS in CRC.

**Abstract:**

Colorectal cancer (CRC) remains a leading cause of cancer-related mortality globally, with significant public health concerns. This review examines the landscape of KRAS inhibition in colorectal cancer (CRC), focusing on recent advances in therapeutic strategies targeting this oncogene. Historically deemed undruggable due to its complex structure and essential role in tumorigenesis, *KRAS* mutations are prevalent in CRC and are associated with poor prognosis. However, breakthroughs in drug development have led to the emergence of KRAS inhibitors as promising treatment options. This review discusses various classes of KRAS inhibitors, including covalent and non-covalent inhibitors, as well as combination therapies aimed at enhancing efficacy and overcoming resistance mechanisms. It highlights recent clinical trials evaluating the efficacy of KRAS inhibitors either as monotherapy or in combination with other agents, such as anti-EGFR antibodies. Despite challenges such as resistance mechanisms and tumor heterogeneity, the development of KRAS inhibitors represents a significant advance in CRC treatment and holds promise for improving patient outcomes in the future.

## 1. Introduction

Colorectal cancer (CRC) is a challenge in oncology, ranking among the most prevalent and lethal malignancies worldwide, in which metastases occur frequently at diagnosis or during disease course. Mutations in the Kirsten rat sarcoma viral oncogene homolog (*KRAS*) gene are key to the pathogenesis of CRC and occur in approximately 40–50% of cases [1]. These mutations confer constitutive activation of *KRAS* signaling pathways, driving tumorigenesis, tumor growth, survival, metastasis, and therapeutic resistance. In addition to CRC, *KRAS* mutations are implicated in various other cancers, including pancreatic cancer (PDAC), lung cancer, and certain subtypes of ovarian and endometrial cancers [2,3,4].

While the advent of targeted therapies has revolutionized the landscape of cancer treatment, precision medicine in the context of CRC remains limited. Around 33% of CRC patients will encounter metastases either at the time of diagnosis or during the course of their illness [5]. Current treatment paradigms for metastatic CRC (mCRC) typically involve a multimodal approach, incorporating mostly chemotherapy and targeted therapies. Chemotherapy regimens, such as FOLFOX (folinic acid, fluorouracil, and oxaliplatin) and FOLFIRI (folinic acid, fluorouracil, and irinotecan), that have long been the cornerstone of mCRC treatment, offer modest improvements in survival outcomes [6,7]. The 5-year relative overall survival (OS) for individuals diagnosed with mCRC is roughly 15% [8]. New compounds have been approved in recent years for the treatment of metastatic colorectal cancer, increasing the median survival of patients from 12 to over 40 months (particularly in *KRAS*wt patients). However, in the case of *RAS* mutation, anti-EGFRs cannot be used, which reduces the therapeutic options, even though the prognosis for these cancers is worse. Despite these improvements, the management of *KRAS*-mutant mCRC remains a huge clinical challenge, and there exists an unmet clinical need for novel therapeutic strategies specifically targeting *KRAS*-mutant mCRC.

Targeting *KRAS* has been a major focus of cancer research over the past four decades. Research and development efforts have intensified over the past decade, largely due to the fundamental discovery by Ostrem et al. of a KRAS G12C inhibitor [9], which is now clinically validated as sotorasib [10,11], offering the potential to directly target mutant KRAS proteins and disrupt aberrant signaling pathways. Additionally, combination strategies incorporating KRAS inhibitors with other targeted agents, immunotherapies, and chemotherapy regimens hold promise in overcoming therapeutic resistance and improving outcomes. This comprehensive review aims to decipher the interplay between *KRAS* signaling and colorectal cancer, exploring the underlying mechanisms driving tumorigenesis, metastasis, and therapeutic resistance. By synthesizing data from preclinical and clinical studies, we endeavor to evaluate the current landscape of KRAS inhibition in mCRC, assessing the clinical efficacy, safety profile, and challenges associated with KRAS-targeted therapies.

## 2. KRAS Pathway

**Figure 1:**
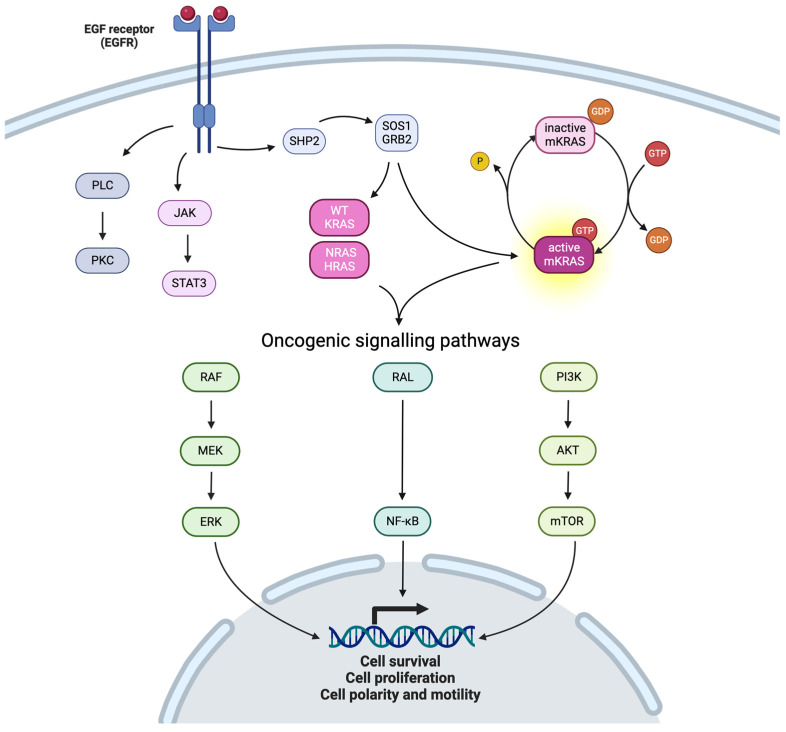
KRAS pathway (created with biorender).

The RAS family consists of three isoforms: HRAS; NRAS; and KRAS. They are cytoplasmic proteins of the GTPase family that play a crucial role in transmitting signals initiated by transmembrane receptors. The KRAS protein acts as a switch for a multitude of cellular signaling functions. The balance between nucleotide hydrolysis and exchange determines the levels of active KRAS in the cells [12]. Bound to GDP, KRAS is in an “OFF” state. Upon the exchange of GDP to GTP, typically in response to growth factors and facilitated by guanine nucleotide exchange factors (GEFs) such as SOS1/SOS2, KRAS transitions to the activated “ON” state. In this form, KRAS activates effector pathways, notably the MAPK and PI3K pathways, to promote cell proliferation and survival. KRAS returns to the “OFF” state when GTP is hydrolyzed to GDP, a process catalyzed by GTPase activating proteins (GAPs) such as NF1 [13]. Most mutations in *KRAS* described in PDAC leave KRAS primarily in the active ON state because GTP hydrolysis is impaired [14,15] (Figure 1).

Activation of RAS by the tyrosine kinase activity of a transmembrane receptor leads to the phosphorylation of RAF and its activation [14]. This triggers a cascade of activation involving the phosphorylation of MEK1/2 (MAPK-ERK Kinase), which, in turn, phosphorylates the ERK family (Extracellular Signal-Regulated Kinase). ERK is then translocated into the nucleus where it activates the transcription of target genes. The RAS/RAF/MAPKinases pathway promotes the expression of genes involved in cell proliferation and cell survival in the epithelial–mesenchymal transition (EMT) and interacts with other signaling pathways to regulate cell motility, including the expression of matrix metalloproteinases (MMPs) and the activity of GTPases proteins [16]. One of these cell pathways is driven by YAP1, which converges with KRAS on the transcription factor FOS to regulate EMT [17]. It was found that YAP1 substitutes for the loss of oncogenic KRAS in human cancers and that YAP1 expression is required for KRAS-induced cell transformation.

**Figure 2:**
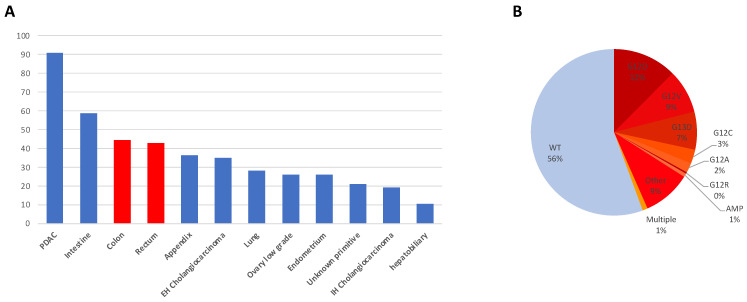
*KRAS* mutations prevalence in solid tumors and CRC. (**A**) Percentage of *KRAS* alterations (tumors with prevalence > 10%), according to the GENIE cohort v13.0-public (red: colorectum) [4]. (**B**) *KRAS* mutation prevalence in CRC (from [1]).

CRC is a cancer that frequently presents a *KRAS* alteration [4] (Figure 2A). However, *KRAS* mutations are diverse, contributing significantly to tumorigenesis and therapeutic resistance [18]. The variety of *KRAS* mutations observed in CRC can be classified based on the type of nucleotide substitution and the affected codon within the *KRAS* gene (Figure 2B). Key types of *KRAS* mutations found in CRC are [19,20]:

1. Point mutations: Point mutations involve the substitution of a single nucleotide base within the *KRAS* gene, resulting in amino acid alterations in the KRAS protein. The most common point mutations in CRC affect codons 12, 13, and 61 of the *KRAS* gene. Codon 12 mutations, such as G12D and G12V, are particularly prevalent, accounting for approximately 50% of *KRAS* mutations in CRC (respectively 28% and 19% for G12D and G12V). G13D is another frequent point mutation (17%) [1]. Codon 61 mutations: mutations at codon 61 of the *KRAS* gene, such as Q61H and Q61L, are less common but still significant in CRC. These mutations account for approximately 5–10% of *KRAS* mutations in CRC and are associated with aggressive tumor phenotypes and resistance to targeted therapies;
2. Insertions and deletions or amplifications: Insertions and deletions (indels) in the *KRAS* gene can lead to frameshift mutations, disrupting the reading frame and generating aberrant KRAS protein variants while less frequent compared to point mutations, as well as amplifications of *KRAS*;
3. Rare mutations: In addition to the commonly observed mutations, CRC may harbor rare or uncommon *KRAS* mutations that involve other codons or unusual nucleotide changes. While individually rare, these mutations collectively contribute to the complexity of KRAS-driven tumorigenesis.

*KRAS* alterations have long been considered “undruggable”, i.e., not targetable, for several reasons [14,21]: (i) The binding affinity between *KRAS* and cytoplasmic GTP is extremely high and limits the possibilities of competitive inhibition; (ii) Intracellular GTP concentrations are high; (iii) *KRAS* lacks a structurally accessible drug-binding pocket on the protein; (iv) There are numerous regulators upstream and downstream of *KRAS* signaling, with signal redundancies that promote resistance to anti-KRAS therapies. The paradigm from considering *KRAS* as undruggable to the development of KRAS inhibitors represents a significant paradigm shift in cancer therapeutics and a promising strategy in CRC.

## 3. KRAS Inhibition: KRAS G12C

### 3.1. Alone

In August 2018, sotorasib (AMG510) became the first KRASG12C inhibitor to enter human clinical trials and demonstrated its safety and clinical efficacy. Hong and colleagues reported the results of a phase I trial [10], multicenter, open-label, testing sotorasib in patients with advanced solid tumors (locally advanced or metastatic) carrying the *KRASG12C* mutation. Overall, 129 patients (59 patients with lung cancer, 42 patients with colorectal cancer, and 28 patients with other types of tumors) were included in the dose escalation and expansion cohorts. Enrolled patients had received a median of three (ranging from 0 to 11) previous lines of anticancer treatments for their metastatic disease. Sotorasib was administered orally once daily, with each treatment cycle lasting 21 days.

The primary endpoint was the incidence of dose-limiting toxic effects (defined as sotorasib-related toxic effects within the first 21 days after the first dose), adverse events during the treatment period, and treatment-related adverse events. Overall, 73 patients (56.6%) experienced treatment-related adverse events, and 15 patients (11.6%) experienced grade 3 or 4 adverse events. The most common grade 3 treatment-related adverse events were gastrointestinal or hepatic effects. Clinical efficacy was investigated as a secondary endpoint. The results were confirmed in phase II, and specific results for colorectal cancer patients are presented in Table 1 [22].

Another inhibitor, adagrasib (MRTX849), has also been tested in phase I and II trials. Preliminary safety data from the KRYSTAL-1 study involving patients with solid tumors treated with adagrasib have been reported [11,23,24,25]. The most frequently reported treatment-related adverse events (>20%) include diarrhea (58%), nausea (52%), fatigue (42%), and vomiting (36%). Among the evaluable colorectal cancer patients for clinical activity, there were 23% confirmed objective responses, while 86% achieved disease control. Additionally, partial responses were confirmed in one patient with endometrial cancer and another patient with pancreatic cancer [11].

Adagrasib and sotorasib are both selective covalent inhibitors of KRASG12C, but pharmacological differences between the two drugs have been reported, including half-life (5 h for sotorasib and 23 h for adagrasib), dose-dependent exposure with adagrasib, and potential penetration into the central nervous system with adagrasib [10,11], interesting in case of brain lesions. New inhibitors are currently under clinical development, such as LY3537982, which has an objective response rate of 42% for PDAC (12 patients), divarasis (7 patients), or in preclinical stages (GDC-6036, D-1553, or garsorasis, JDQ443, BI 1823911) [26].

**Table 1:** Safety and toxicity data regarding KRASG12C inhibitors in CRC.

	Adagrasib (*n* = 43) [24]	Sotorasib (*n* = 62) [22]
Objective response rate	23% (12–39%)	9.7% (3.6–19.9%)
Disease control rate	86%	82.3% (70.5–90.8)
Median duration of response	4.3 months (2.3–4.4)	4.2 months (2.9–8.5)
Progression-free survival	5.6 months (4.1–8.3)	4.0 months (2.8–4.2)
Overall survival	19.8 months (12.5–23.0)	10.6 months (7.7–15.6)
Grade 3–4 toxicities	34%	10%
Dose reduction	39%	18%
Discontinuation for toxicity	0%	2%

(): IC95%.

Several resistance mechanisms to anti-KRASG12C have already been described [27]: (i) bypassing signaling pathways with reactivation of the KRAS-MAPK pathway via positive feedback from mediators upstream or downstream of the KRAS-MAPK cascade (Figure 2); (ii) signaling of the KRAS pathway mediated by wild-type forms of RAS (NRAS and HRAS) (Figure 2); (iii) increased proliferation via cell cycle disinhibition with metabolic reprogramming (including glutamine metabolism); and (iv) alteration of the immune response. One of the major regulators for resistance against KRASG12C inhibitors is driven by the Hippo (YAP/TAZ) signaling pathway with nuclear translocation of YAP, providing some potential targets to overcome resistance [28].

### 3.2. Combination with Other Drugs

**Figure 3:**
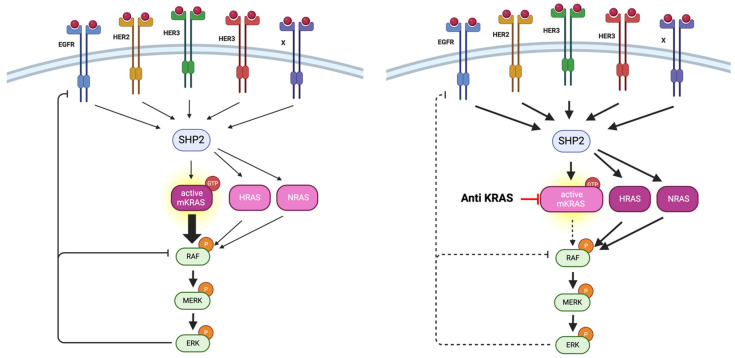
Mechanisms of bypassing KRAS inhibitors through the activation of wild-type (WT) forms of RAS (NRAS and HRAS) [29,31] (biorender).

Although initial data from studies on adagrasib or sotorasib monotherapy [10,11] are promising in patients with *KRASG12C* mutations, reactivation may occur in the RAS-MAPK signaling pathway due to a feedback loop mediated by the epidermal growth factor receptor (EGFR) [29,30] (Figure 3). Activation of wild-type RAS isoforms (NRAS and HRAS) leads to MAPK pathway activation in the presence of the KRASG12C inhibitor [29,30]. Thus, vertical inhibition strategies (double or triple with SHP2, MEK, and/or EGFR inhibitors) may enhance the activity of KRASG12C inhibitors [29,30].

The combination of an EGFR-directed antibody with a KRASG12C inhibitor is a clinically effective strategy to mitigate EGFR reactivation (Table 2). Cetuximab or panitumumab are monoclonal anti-EGFR antibodies indicated in the treatment of metastatic colorectal cancer with wild-type *RAS*, either as monotherapy or in combination with chemotherapy [32,33]. In a recent phase Ib study involving sotorasib in combination with the EGFR-targeted monoclonal antibody panitumumab, an objective response rate of 30% was reported in pretreated patients with colorectal cancer harboring a *KRASG12C* mutation, compared to 10% for sotorasib monotherapy [22,34]. Phase III results were reported at ESMO 2023, with a progression-free survival of 5.6 months in the combination arm [24]. Median OS was not reached for sotorasib 960mg + panitumumab, 11.9 months for sotorasib 240mg + panitumumab, and 10.3 months for the investigator’s choice [35]. Furthermore, in colorectal cancer, the combination of adagrasib and cetuximab was associated with a 46% response rate and a median response duration of 7.6 months. Additionally, the therapeutic combination was associated with a median progression-free survival of 6.9 months, suggesting potential improvement in clinical benefits through this combination approach.

Other combinations have been reported with divarasib (29 patients), garsorasib (29 patients), or LY3537982 (11 patients) with similar results [26].

## 4. Other Strategies to Target *KRAS*

### 4.1. Other Codon Specific Inhibitor: MRTX1133 (KRASG12D)

#### 4.1.1. Preclinical Data

The protein corresponding to the *KRASG12D* gene, unlike the KRASG12C protein, does not contain a residue, allowing for a covalent binding, and involves a different cycle between active and inactive forms [37]. Thus, the development of a KRASG12D inhibitor used a different strategy from that of KRASG12C inhibitors. Through optimization based on the molecular structure of KRAS, the Mirati^®^ team has developed MRTX1133 [38], a selective and potent inhibitor of KRASG12D, which is non-covalent and exhibits picomolar binding affinity to the protein. Using techniques to directly measure molecular binding, MRTX1133 has demonstrated approximately 700 times higher selectivity for binding to KRASG12D compared to wild-type KRAS [38]. Various pharmacological tests have shown that MRTX1133 strongly and selectively binds to both active and inactive forms of KRASG12D.

The MRTX1133 was then evaluated in cellular assays to assess its effect on KRAS signaling and cell viability [39]. Following a three-hour treatment on mutant *KRASG12D* cancer cell lines HPAC (pancreatic) and GP2D (colorectal), MRTX1133 caused inhibition (concentration-dependent) of key signaling molecules in the KRAS pathway, including phospho-ERK (pERK) [38,39], with IC50 values ranging from 0.6 nM to 13.7 nM (median IC50: 6.1 nM). In contrast, IC50 values ranged from 151 nM to over 3000 nM (median IC50: >3000 nM) in non-mutant *KRASG12D* cell lines. Assessing cell viability, MRTX1133 inhibited both 2D and 3D *KRASG12D* cell lines with IC50 values ranging from 1.4 nM to 42.3 nM (3D) and between 1.5 nM and 299 nM (2D). These data demonstrate that MRTX1133 potently and selectively inhibits KRAS-mediated signaling and viability in the vast majority of mutant *KRASG12D* cancer cell lines (in vitro models).

MRTX1133 was also evaluated in immunocompromised mice bearing tumor xenografts with the *KRASG12D* mutation to determine its effect on KRAS-mediated signaling and characterize its in vivo antitumor activity. MRTX1133 induced tumor regression of 30% or more in 11 out of 25 *KRASG12D* mutant models (PDX or cell lines) [39]. This antitumor activity was particularly significant in pancreatic cancer models, where 8 out of 11 (73%) models exhibited tumor regression of 30% or more [39]. Another study showed that alongside tumor cell apoptosis and proliferation arrest, MRTX1133 led to marked changes in the tumor microenvironment, notably affecting fibroblasts, matrix, and macrophages [40]. T cells were necessary for MRTX1133 to have a complete antitumor effect, and T cell depletion accelerated tumor regrowth after treatment [40].

#### 4.1.2. Combination Perspectives

##### EGFR

A few recent studies have validated the interest of combining MRTX1133 with anti-EGFR agents (all published in 2023) [31,41]. While treatment with MRTX1133 led to marked antitumor activity in most tested models, a subset of models was less sensitive to MRTX1133 and showed either tumor growth inhibition or stable disease as the best response. Additional data obtained using a CRISPR-Cas9 screen suggested that the combined targeting of EGFR, PI3Kα (encoded by the PIK3CA gene), or SHP2 (encoded by the PTPN11 gene) could complement KRASG12D inhibition and represent therapeutic strategies to enhance MRTX1133 activity [39].

One study also reports that MRTX1133 induces reversible growth arrest of mutant *KRASG12D* CRC cells, accompanied by partial reactivation of RAS effector signaling [31], and it has been shown that EGFR inhibition is synthetically lethal with MRTX1133. Mechanistically, MRTX1133 reduces ERRFI1 expression, a crucial negative regulator of EGFR, leading to retroactive activation of EGFR. Notably, wild-type RAS isoforms, including HRAS and NRAS, but not the oncogenic KRAS, transmit downstream signaling from activated EGFR, resulting in rebound signaling of RAS effectors and reduced efficacy of MRTX1133. Blocking activated EGFR with antibodies or tyrosine kinase inhibitors suppresses this wild-type EGFR/RAS signaling axis, thus sensitizing cells to MRTX1133 (organoid or xenograft models) [31].

Another study shows that MRTX1133 increased the expression and phosphorylation of EGFR (ERBB1) and HER2 (ERBB2) [41]. The use of an irreversible pan-ERBB inhibitor, afatinib, strongly synergized with MRTX1133 in vitro, and cancer cells with acquired resistance to MRTX1133 in vitro became sensitive again under this therapeutic combination. Finally, the combination of MRTX1133 and afatinib resulted in tumor regression and longer survival in murine orthotopic PDAC models [41].

##### Immune Checkpoint Inhibitors

The management of tumors is therapeutically complicated by the tumor microenvironment (TME), which includes immune cells, cancer-associated fibroblasts (CAFs), and a dense extracellular matrix [42]. These characteristics are replicated in a genetically PDAC-modified mouse model that incorporates KRASG12D and TP53R172H mutations (KPC), in which tumor cells can be identified by a YFP lineage marker (KPC/Y) [43,44]. It is noteworthy that KPC/Y mice bearing tumors are refractory to most therapeutic interventions [45], which makes this model useful for prioritizing therapeutic candidates and defining their mechanisms of action in an immunocompetent environment. This KPC/Y model and several of its clonal derivatives have been tested to evaluate the efficacy and biological impact of MRTX1133 [40]. The authors demonstrated that MRTX1133 led to a decrease in cell proliferation and an increase in cell death early in the treatment, inducing tumor regression [40]. It was also shown that the drug causes changes in the TME, which may contribute to the antitumor effect, including an increase in tumor-associated macrophages (adopting an M1-like phenotype) and αSMA+ myofibroblasts (types of cells known for their tumor-inhibitory properties). MRTX1133 also induced increased infiltration of tumor T cells and a role of T cell-mediated immunity in achieving deeper tumor regressions and sustained disease control.

These observations support the interest in combining MRTX1133 with immunotherapy (immune checkpoint inhibitors, tumor-infiltrating lymphocytes (TIL), or CAR T-cell therapy), stimulating the patient’s immune system to work with the drug to achieve complete and durable tumor eradication, and could lead to specific therapeutic trials [46,47].

### 4.2. Other Therapeutic Class

In recent years, there has been the emergence of three major classes of anti-KRAS drugs: drugs selectively targeting mutant variants of *KRAS* (G12C, G12D, etc.) as described above; pan-KRAS inhibitors targeting a wide and diverse spectrum of *KRAS* alterations, covering mutations and amplifications; and indirect inhibitors of KRAS (SOS1, SHP2) [12,14,19,21,26].

Last, cancer metabolism could be used in *KRAS*-driven CRC. Indeed, *KRAS* mutant CRC harbors a boosted glucose metabolism as well as deregulation of glutamine, amino-acid, and fatty acid metabolism that support cancer cell proliferation [48]. This rewired metabolism is a putative therapeutic target for the treatment of *KRAS* mutant CRC or to overcome therapeutic resistances, with pormising responses in vitro [48] and phase 1 or 2 ongoing [28].

#### 4.2.1. Pan-KRAS Inhibitors

Using fragment-based screening of KRAS and structure-based drug design, Boehringer Ingelheim^®^ recently announced the discovery of pan-specific direct KRAS inhibitors and proteolysis-targeting chimeras (PROTACs) targeting KRAS, capable of sparing NRAS and HRAS [1]. This new emerging class of drugs (PROTAC) specifically degrades proteins via the cellular protein degradation system [49]. These molecules interact simultaneously with a protein of interest and an E3 ligase, forming a ternary complex that allows the E3 ligase to ubiquitinate and induce degradation of the target protein [50].

It also appears possible to target a broad spectrum of *KRAS* mutations through various pan-RAS inhibitors that block all three RAS isoforms: KRAS; NRAS; and HRAS. The pan-RAS strategy adopted by Boehringer Ingelheim^®^ focuses on inhibitors of the switch I/II pocket, such as the compound BI-2865 [51,52]. This non-covalent inhibitor preferentially binds to and with high affinity to the inactive state of *KRAS* while preserving NRAS and HRAS HRAS [52]. It blocks nucleotide exchange to prevent the activation of wild-type *KRAS* as well as a wide range of *KRAS* mutations, including G12A/C/D/F/V/S, G13C/D, V14I, L19F, Q22K, D33E, Q61H, K117N, and A146V/T. Inhibition of downstream signaling and proliferation is limited to cancer cells carrying a *KRAS* mutation, and treatment with the drug prevents the growth of mutant *KRAS* tumors in mice without any detrimental effect on animal weight.

Revolution Medicines^®^ used another molecular binding approach to discover RMC-6236, which is described as a potent RAS inhibitor that is available orally [53]. The preclinical efficacy of these inhibitors has been established [1]. Phase I results were described at ESMO 2023, with a response rate of 20% in patients with PDAC (65 treated patients, 46 evaluable patients). More data are needed for CRC patients (10 CRCs were included in phase I). The most frequent treatment-related adverse events (TRAEs) were rash (maculopapular (13%) or acneiform (35%)), gastrointestinal-related toxicities (nausea (32%), and diarrhea [19%]) and were mostly grade 1 or 2. It remains to be established whether simultaneous targeting of all three RAS isoforms will be compatible with achieving a therapeutic window in patients (i.e., with manageable toxicities), but the initial data from the phase I trial are reassuring, with mainly the rash and gastrointestinal disorders.

#### 4.2.2. Indirect Inhibition

Several drugs targeting *KRAS* indirectly are currently in development by interfering with nucleotide exchange and *KRAS* activation via SHP2 or SOS1 inhibition [54,55,56,57,58]. The rationale for SHP2 and SOS1 inhibitors as pan-KRAS inhibitors relies on the *KRAS* cycle between an inactive and active state, which is dependent on (i) upstream activation and (ii) nucleotide exchange.

SHP2 inhibitors stabilize an inhibited enzyme conformation and, thus, disrupt the nucleotide exchange of KRAS mediated by SOS1 [56,59,60]. SHP2 inhibitors, such as RMC-4630, TNO155, and JAB-3068, have reached phase II clinical trials. In a phase I/II study (NCT03634982) with RMC-4630, including patients with tumors harboring *RAS* alterations (including *KRAS* amplification), initial clinical data showed a disease control rate of 71% (5/7 patients) with tumor volume reduction observed in three patients (43%) and a confirmed objective response in one patient with *KRASG12C*-mutated lung cancer [61]. Initial clinical data in a phase I study (NCT03114319) with the SHP2 inhibitor TNO155 showed sensitivity in some tumors mutated in *KRASG12*, particularly *KRASG12C*-mutated lung cancer and *BRAF/NRAS* mutation-negative melanoma, but no partial responses [62].

SOS1 inhibitors block the interaction between SOS1 and KRAS-GDP, preventing nucleotide exchange and GTP loading of KRAS [57]. BI-1701963 is currently the only SOS1 inhibitor under clinical trials. BI-1701963 has been generally well tolerated, with a maximum tolerated dose reached at 800 mg, and stable disease for up to 18 weeks demonstrated in 7 out of 31 patients with solid tumors harboring KRAS mutations [63].

SOS1 and SHP2 inhibitors also generate interest in therapeutic combinations to determine if vertical blockade of different pathways can “tighten the grip” on the KRAS/MAPK pathway and, thus, increase response rates and duration. SHP2 inhibitors and BI-1701963 are being combined with MEK inhibitors (trials NCT04294160, NCT03989115, NCT04720976, NCT04111458, and NCT048357) to enhance MAPK pathway modulation and suppress pathway reactivation induced by relief of negative feedback control. SHP2 inhibitors are also combined with ERK inhibitors (NCT04916236) and EGFR inhibitors (NCT03989115 and NCT03114319). Since SOS1 and SHP2 inhibitors alter the balance of KRAS toward the GDP-bound state, it would also be logical to combine these indirect KRAS modulators with specific *KRAS* mutation inhibitors, such as covalent inhibitors of KRASG12C that bind to KRAS in its GDP-bound state.

#### 4.2.3. Cancer Vaccines

Several vaccination approaches, such as mRNA and peptide vaccines, are being explored to enhance T cell activation against mutant KRAS neoantigens. A phase 1 clinical study is currently investigating the mRNA-5671/V941 vaccine either alone or in combination with anti-PD-1 (pembrolizumab) in patients diagnosed with advanced or metastatic microsatellite stable CRC harboring one of the four *KRAS* mutations (G12D, G12V, G13D, or G12C) and specific HLA types, including HLA-A11:01 or HLA C08:02 (NCT03948763). Another phase 1 trial is underway, testing a combination of pooled mutant-KRAS peptide vaccine with nivolumab and ipilimumab in microsatellite stable CRC (NCT04117087). TG02, a neoantigen peptide cancer vaccine developed by Targovax, comprises eight synthetic peptides representing fragments of the most common *RAS* mutant peptides observed in rectal cancer. In a phase 1b clinical trial, TG02 was administered alone or in combination with pembrolizumab to patients with locally advanced primary or recurrent KRAS codon 12 or 13 (exon 2) mutant CRC (NCT02933944). TG02 doses were given alongside granulocyte–macrophage colony-stimulating factor prior to pelvic surgery. Among the six enrolled CRC patients, four exhibited a TG02-specific immune response as assessed by delayed-type hypersensitivity, and three showed systemic presence of TG02-specific T cells.

#### 4.2.4. CAR-T Cells

Adoptive cell therapy with the use of T cells engineered to express allogeneic T-cell receptors (TCRs) targeting *KRAS* mutations may be another approach. Some cases are described in which CAR-T cell therapy mediated regression of metastatic solid cancers. Using CD8^+^ T cells that were reactive to *KRASG12D* mutant, a patient with CRC experienced prolonged partial response [64]. In a patient with PDAC, TCR gene therapy targeting *KRASG12D* mutation mediated the objective regression of metastatic lesions [65]. Prospective clinical trials are needed to fully determine the therapeutic opportunity of this CAR-T therapy in cancers harboring *KRAS* mutation, as well as determine the potential toxicities.

**Figure 4:**
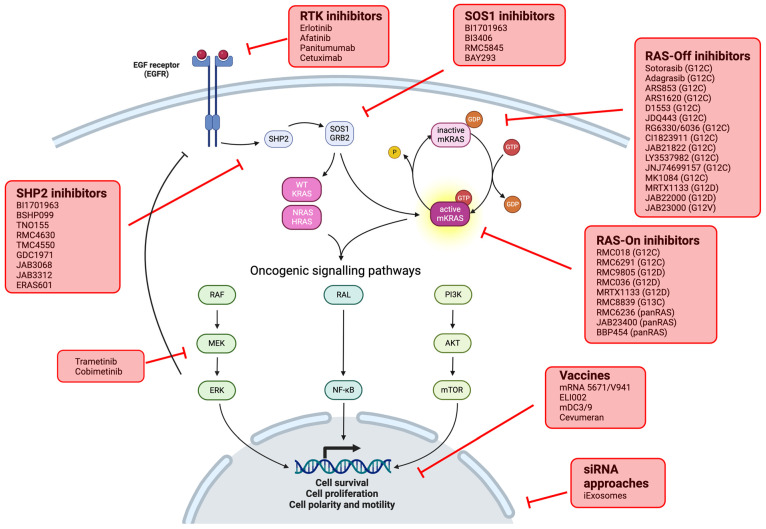
Therapeutic approaches to target KRAS.

Ongoing clinical trials for *KRAS* mutant CRC are described in Table 3 and strategies summarized on Figure 4.

## 5. Conclusions

In conclusion, this review provides a comprehensive overview of the emerging landscape of KRAS-targeted therapies, highlighting the significant progress made in recent years. From the development of selective inhibitors targeting specific KRAS mutations like G12C and G12D to pan-KRAS inhibitors and indirect KRAS modulators, the therapeutic arsenal against *KRAS*-driven cancers is rapidly expanding.

The discovery of inhibitors like sotorasib, adagrasib, MRTX1133, and others has brought new hope for patients with KRAS-mutant tumors, demonstrating promising clinical efficacy and tolerability profiles in early-phase trials. Additionally, the emergence of PROTAC technology offers a novel approach to target KRAS proteins, potentially overcoming challenges associated with traditional small molecule inhibitors. Furthermore, the exploration of combination therapies involving KRAS inhibitors with agents targeting upstream or downstream effectors of the KRAS pathway represents a promising strategy to enhance treatment response and overcome resistance mechanisms. However, challenges remain, particularly in addressing resistance mechanisms and optimizing therapeutic regimens to maximize efficacy while minimizing toxicity. Further clinical studies are warranted to validate the efficacy of these emerging therapies, explore optimal combination strategies, and identify predictive biomarkers to guide patient selection. Overall, the advancements in KRAS-targeted therapies outlined in this review underscore the growing momentum in the field and the potential to revolutionize the treatment landscape for *KRAS*-driven CRC, ultimately improving patient outcomes and quality of life by possibly avoiding toxic chemotherapeutics treatments. In the future, potential combinations of the different approaches highlighted in this manuscript should be explored, especially KRAS inhibitors potentially combined with immunotherapy.

## Figures and Tables

**Table 2:** Results of combination therapy with KRASG12C and EGFR inhibitors in colorectal cancer.

Criteria	Adagrasib + Cetuximab (*n* = 28) [24]	Sotorasib + Panitumumab (*n* = 53) [34,36]
Objective response rate % (CI95%)	46% (28–66)	26.4% (15.3–40.3)
Median duration of response Months (CI95%)	7.6 (5.0–NR)	4.4 (2.8–6.3)
Median progression-free survival Months (CI95%)	6.9 (5.4–8.1)	5.6 (4.2–7.6)
Median overall survival Months (CI5%)	13.4 (9.5–20.1)	NR (9.6–NR) (960 mg) and 11.9 (7.5–NR) (240 mg)

CI: confidence interval; NR: non-reached.

**Table 3:** Ongoing trials targeting KRAS mutant CRC.

NCT Number	Drug	Target	Main Sponsor	Study Status
Specific inhibitor alone				
NCT05485974	HBI-2438	KRASG12C	HUYABIO International, LLC. (San Diego, CA, USA)	RECRUITING
NCT05462717	RMC-6291	KRASG12C	Revolution Medicines, Inc. (Redwood City, CA, USA)	ACTIVE_NOT_RECRUITING
NCT06244771	FMC-376	KRASG12C	Frontier Medicines Corporation (San Fransisco, CA, USA)	RECRUITING
NCT04121286	JAB-3312	KRASG12C	Jacobio Pharmaceuticals Co., Ltd. (Beijing, China)	RECRUITING
NCT06385925	TSN1611	KRASG12D	Tyligand Bioscience (Shanghai, China)	RECRUITING
NCT05737706	MRTX1133	KRASG12D	Mirati Therapeutics Inc. (San Diego, CA, USA)	RECRUITING
NCT06364696	ASP4396	KRASG12D	Astellas Pharma Inc.(Tokyo, Japan)	RECRUITING
NCT06403735	QLC1101	KRASG12D	Qilu Pharmaceutical Co., Ltd. (Madrid, Spain)	RECRUITING
NCT06040541	RMC-9805|RMC-6236	KRASG12D/KRASG12X	Revolution Medicines, Inc. (Redwood City, CA, USA)	RECRUITING
Specific inhibitor in combinaiton				
NCT06412198	Cetuximab|Cemiplimab|Adagrasib	KRASG12C	M.D. Anderson Cancer Center (Houston, TX, USA)	RECRUITING
NCT05194995	JAB-21822|Cetuximab	KRASG12C	Jacobio Pharmaceuticals Co., Ltd. (Beijing, China)	ACTIVE_NOT_RECRUITING
NCT04330664	MRTX849|TNO155	KRASG12C + SHP2	Mirati Therapeutics Inc. (San Diego, CA, USA)	ACTIVE_NOT_RECRUITING
NCT05288205	JAB-21822|JAB-3312	KRASG12C + SHP2	Jacobio Pharmaceuticals Co., Ltd. (Beijing, China)	RECRUITING
NCT06039384	INCB099280|adagrasib	KRASG12C	Incyte Corporation (Geneva, Switzerland)	ACTIVE_NOT_RECRUITING
NCT05198934	Sotorasib|Standard treatment	KRASG12C	Amgen (Thousand Oaks, CA, USA)	ACTIVE_NOT_RECRUITING
NCT04956640	LY3537982|standard treamtent	KRASG12C	Eli Lilly and Company (Indianapolis, IN, USA)	RECRUITING
NCT05578092	MRTX0902|MRTX849	KRASG12C + SOS1	Mirati Therapeutics Inc. (San Diego, CA, USA)	RECRUITING
NCT04699188	JDQ443|TNO155	KRASG12C +SHP2	Novartis Pharmaceuticals (Reuil Malmaison, France)	RECRUITING
NCT04449874	GDC-6036|Standard treamtent	KRASG12C	Genentech, Inc. (San Franscisco, CA, USA)	RECRUITING
NCT05358249	JDQ443|trametinib|Ribociclib	KRASG12C	Novartis Pharmaceuticals (Reuil Malmaison, France)	ACTIVE_NOT_RECRUITING
NCT06026410	KO-2806|Cabozantinib|Adagrasib	KRASG12C, farnesyl transferase	Kura Oncology, Inc. (San Diego, CA, USA)	RECRUITING
NCT06252649	Sotorasib|standard treatment	KRASG12C	Amgen (Thousand Oaks, CA, USA)	RECRUITING
NCT06586515	LY3962673|standard treatment	KRASG12D	Eli Lilly and Company (Indianapolis, IN, USA)	NOT_YET_RECRUITING
NCT04793958	MRTX849|standard treatment	KRASG12D	Mirati Therapeutics Inc. (San Diego, CA, USA)	ACTIVE_NOT_RECRUITING
NCT03785249	MRTX849|standard treatment	KRASG12D	Mirati Therapeutics Inc. (San Diego, CA, USA)	RECRUITING
NCT05722327	MRTX849|standard treatment	KRASG12D	M.D. Anderson Cancer Center (Houston, TX, USA)	RECRUITING
NCT06599502	AZD0022|Cetuximab	KRASG12D	AstraZeneca (London, UK)	NOT_YET_RECRUITING
panKRAS				
NCT06078800	YL-17231	KRAS	Shanghai YingLi Pharmaceutical Co., Ltd. (Shanghai, China)	RECRUITING
NCT06607185	LY4066434|standard treatment	KRAS	Eli Lilly and Company (Indianapolis, IN, USA)	NOT_YET_RECRUITING
NCT06447662	PF-07934040|standard treatment	KRAS	Pfizer (New York, NY, USA)	RECRUITING
NCT06585488	BGB-53038|Tislelizumab|Cetuximab	KRAS	BeiGene (Beiging, China)	NOT_YET_RECRUITING
NCT06445062	RMC-6236|Standard treatment	KRAS	Revolution Medicines, Inc. (Redwood City, CA, USA)	RECRUITING
NCT05379985	RMC-6236	KRAS	Revolution Medicines, Inc. (Redwood City, CA, USA)	RECRUITING
RAF/MEK				
NCT05786924	BDTX-4933	RAF	Black Diamond Therapeutics, Inc. (Cambridge, MA, USA)	RECRUITING
NCT06194877	BGB-3245|Panitumumab	RAF	MapKure, LLC (Stamford, CT, USA)	RECRUITING
NCT05200442	VS-6766	RAF/MEK	University of Chicago (Chicago, IL, USA)	RECRUITING
NCT06270082	IK-595	MEK/RAF	Ikena Oncology (Boston, MA, USA)	RECRUITING
NCT05163028	HBI-2376	SHP2	HUYABIO International, LLC. San Diego, CA, USA)	RECRUITING
Cancer vaccines				
NCT04117087	KRAS peptide vaccine|Nivolumab|Ipilimumab	KRAS vaccine	Sidney Kimmel Comprehensive Cancer Center at Johns Hopkins (Adelaide, Australia)	RECRUITING
NCT04853017	ELI-002 2P	KRAS vaccine	Elicio Therapeutics (Boston, MA, USA)	ACTIVE_NOT_RECRUITING
NCT06411691	KRAS Vaccine|Balstilimab|Botensilimab	KRAS vaccine	Sidney Kimmel Comprehensive Cancer Center at Johns Hopkins (Adelaide, Australia)	NOT_YET_RECRUITING
NCT05726864	ELI-002 7P	KRAS vaccine	Elicio Therapeutics (Boston, MA, USA)	RECRUITING
CART				
NCT06105021	AFNT-211	CART	Affini-T Therapeutics, Inc. (Boston, MA, USA)	RECRUITING
NCT06253520	KRAS TCR-Transduced PBL	CART	National Cancer Institute (NCI) (Bethesda, MD, USA)	RECRUITING
NCT06487377	IX001 TCR-T cells	CART	Shanghai Pudong Hospital (Shanghai, China)	RECRUITING
NCT06218914	NT-112	CART	Neogene Therapeutics, Inc. (Santa Monica, CA, USA)	RECRUITING
